# Association of Immigrant and Refugee Status With Risk Factors for Exposure to Violent Assault Among Youths and Young Adults in Canada

**DOI:** 10.1001/jamanetworkopen.2020.0375

**Published:** 2020-03-04

**Authors:** Natasha Ruth Saunders, Jun Guan, Alison Macpherson, Hong Lu, Astrid Guttmann

**Affiliations:** 1 Division of Pediatric Medicine, The Hospital for Sick Children, Toronto, Ontario, Canada; 2 Department of Pediatrics, University of Toronto, Toronto, Ontario, Canada; 3 ICES, Toronto, Ontario, Canada; 4 Child Health Evaluative Sciences, SickKids Research Institute, Toronto, Ontario, Canada; 5 Institute of Health Policy, Management and Evaluation, The University of Toronto, Toronto, Ontario, Canada; 6 York University, Toronto, Ontario, Canada

## Abstract

**Importance:**

Immigrant populations continue to grow across Western countries. Such populations may face vulnerabilities that contribute to the risk of experiencing violent injury. Youths are at disproportionate risk compared with other age groups, and such violence may be preventable with appropriately targeted injury prevention strategies.

**Objective:**

To examine the association of immigrant or refugee status and immigration-related factors with the experience of assault.

**Design, Setting, and Participants:**

This population-based cohort study used linked health and administrative databases in Ontario, Canada, where health services are funded through a universal, single-payer health insurance plan. All youths and young adults aged 10 to 24 years (hereafter referred to as *youths*) residing in Ontario from January 1, 2008, to December 31, 2016, were eligible to participate. Data were analyzed from April 13, 2017, to January 6, 2020.

**Exposures:**

The main exposure was immigrant status. Secondary exposures were immigration-related factors, including visa class, time since immigration, and region and country of origin.

**Main Outcomes and Measures:**

The main outcome consisted of violent injuries requiring acute care (emergency department visit or hospitalization) or causing death. Poisson regression models were used to estimate rate ratios for injuries.

**Results:**

A total of 22 969 443 person-years were included in the analysis (51.3% male and 48.7% female participants). Compared with nonimmigrants, a greater proportion of immigrants lived in the lowest neighborhood income quintile (30.5% vs 18.2%) and urban areas (98.9% vs 87.7%). Among immigrants, 17.9% were refugees. Rates of violent injuries experienced were 549.0 (95% CI, 545.7-552.2) per 100 000 person-years in nonimmigrant youth, 225.0 (95% CI, 219.4-230.7) per 100 000 person-years in nonrefugee immigrant youth, and 525.4 (95% CI, 507.2-544.1) per 100 000 person-years in refugee immigrant youth. The rates of violent injury among nonrefugee and refugee immigrants were lower than among nonimmigrants (nonrefugee adjusted rate ratio [aRR], 0.41 [95% CI, 0.38-0.43]; refugee aRR, 0.82 [95% CI, 0.76-0.89]). Older age (oldest vs youngest aRR, 6.90 [95% CI, 6.53-7.29]), male sex (aRR, 2.60 [95% CI, 2.52-2.68]), and low neighborhood income (aRR, 2.42 [95% CI, 2.32-2.53]) were associated with violent injury risk. Rates of experiencing assault were lowest among South Asian (aRR, 0.33 [95% CI, 0.30-0.37]) and East Asian (aRR, 0.23 [95% CI, 0.19-0.26]) immigrants. Only Somali immigrants experienced higher assault rates (712.0 [95% CI, 639.3-805.3] per 100 000 person-years) compared with nonimmigrants. Most injuries (79.9%) were from being struck, followed by being cut (5.9%).

**Conclusions and Relevance:**

The low rates of assault experienced by immigrants, including refugees, compared with nonimmigrants suggests that Canadian immigrant settlement supports and cultural factors may be protective against the risk of experiencing assault.

## Key Points

**Question:**

What is the risk of experiencing violent injury associated with immigrant and refugee status among youth and young adults in Canada?

**Findings:**

In this population-based cohort study including 22 969 443 person-years, the adjusted risk ratio of experiencing assault among immigrants was 0.41 and among refugees was 0.82 compared with nonimmigrant individuals. Risk of assault among immigrants was stable with time since immigration, and rates were lowest among immigrants from South and East Asia.

**Meaning:**

The low relative rates of assault among immigrants suggest that Canadian immigrant settlement supports and cultural factors may be protective against the risk of experiencing assault.

## Teaser

This cohort study examines the association of immigrant or refugee status and immigration-related factors with the experience of assault among youths and young adults in Ontario, Canada.

## Introduction

Violence against youth is a global phenomenon and a leading cause of death in high-income countries.^1,2^ Prevalence rates vary considerably depending on the country, culture, and socioeconomic climate. Youths, in particular, are a group who disproportionately experience violence, with reported rates approximately 4 times higher than for middle-aged adults (aged 35-64 years) and 15 times higher than for adults 65 years or older.^3^ Physical violence in youth, which is detrimental to physical and mental well-being,^2^ may stem from punishment from caregivers, assault from strangers, bullying, and criminal or gang involvement. Understanding the distribution of youth who die due to or who experience and survive physical violence by sociodemographic factors, including immigration factors, can guide interventions and policies for violence prevention efforts.

Foreign-born individuals now constitute 21.9% of the Canadian population.^4^ Immigrants to Canada come from more than 150 source countries for 1 of 3 main reasons: family reunification (ie, family class immigrants), humanitarian or compassionate needs (ie, refugees), and the ability to contribute economically and fill labor market needs (ie, economic class immigrants).^4^ As the number of immigrants continues to rise in Canada and most high-income countries,^5^ public concern has increased about racism, marginalization, poverty, use of public resources, criminal gang involvement, and violence among immigrant youth.^6,7,8,9^ Increasing global migration with a simultaneous rise in urban violence has fueled anti-immigrant sentiment, and the role immigrants play in contributing to this violence has dominated discourse.^10,11,12^ Given the lack of public crime statistics by immigrant status, conclusions cannot be drawn regarding causal relationships between immigration and violence.

Immigrant experiences before and after migration may affect the risk of experiencing violence after resettlement. Immigrant youth, in particular refugees (those who typically have undergone forced migration), are vulnerable, often facing social disadvantage and challenged to adapt to new cultural milieus, language, social structures, and peer relationships. Immigrant youth who migrate with their families or to join them may experience discrimination, racism, xenophobia, bullying, peer aggression, and family or gang violence. These experiences may limit social and educational opportunities and leave lasting physical and emotional injury, which in turn may limit a youth’s ability to realize his or her potential.^13,14,15^ Understanding how migration factors affect the risk of experiencing violent injury is critical for the development and implementation of targeted injury prevention and settlement strategies. However, objective data on violence experienced by immigrant youths that do not rely on self-report, globally and in a Canadian context, are notably absent from existing published work to date.

Our objectives were to describe at a population level the rates of serious assault (ie, requiring a hospital visit) in immigrant and nonimmigrant youths and young adults (hereafter referred to as *youths*) in Ontario, Canada, and to test the association of immigrant status and immigration-related factors with the experience of violent injury. We hypothesized that rates of experiencing violence are lower among immigrant youths, but the distribution patterns of experiencing violence vary between regions and countries of origin as well as by refugee status and time since migration.

## Methods

### Study Design, Setting, and Population

This population-based cohort study used health and administrative data sets linked at ICES, a not-for-profit research institute whose legal status under Ontario’s health information privacy law allows it to collect and analyze health data without consent in Toronto, Canada. This study was approved by the research ethics board at The Hospital for Sick Children, Toronto, Ontario, and followed the Strengthening the Reporting of Observational Studies in Epidemiology (STROBE) and Reporting of Studies Conducted Using Observational Routinely Collected Data (RECORD) reporting guidelines.

In Ontario, the single-payer, universal Ontario Health Insurance Plan provides publicly funded health care for most hospital and physician services at no personal cost to all Ontarians, including immigrants with permanent residency in Canada. We included all youths aged 10 to 24 years living in Ontario from January 1, 2008, to December 31, 2016, with a valid Ontario Health Insurance Plan number. Nine annual cohorts were created within the study period to assign sociodemographic characteristics based on data available on December 31 of each cohort year.

### Data Sources

A unique, encoded identifier for each individual, derived from Ontario Health Insurance Plan numbers, was used to link several data sets. We extracted and linked data from the Registered Persons Database (Ontario’s health insurance registry), the National Ambulatory Care Reporting System (emergency department visit data), the Canadian Institute for Health Information Discharge Abstract Database (hospitalization data), the Ontario Registrar General Database (deaths data),^16^ and the Rurality Index of Ontario^17^ based on postal code (rural vs urban residence). Statistics Canada’s Postal Code Conversion File was used to determine neighborhood income quintiles at the dissemination area level using Canadian Census data from 2006. A detailed outline of the data linkage process has been published elsewhere.^16^

To obtain immigration information, we used the Immigration, Refugees and Citizenship Canada Permanent Resident Database. The available portion of this database contains data for all permanent residents (immigrants granted permission to live and work in Canada without limitations on their stay) landing in Ontario since 1985. Deterministic and probabilistic linkage to the Registered Persons Database identifies 86% of permanent residents.^16^ Not included in this data set are undocumented or temporary residents (eg, foreign students) and prehearing asylum seekers (including those who have not yet obtained residency status but eventually do). Permanent residents, including refugees, linked in the database are typically eligible for provincial health insurance within 3 months of arrival in Canada.

### Outcome Measures

The primary outcome measure was any serious assault-related injury. Serious assaults were defined as any emergency department visit, hospital admission, or in- and out-of-hospital death (eg, homicides) using the *International Statistical Classification of Diseases and Related Health Problems, Tenth Revision, Canada,* codes for external causes of injury with a discharge diagnosis of an intentional injury (X85-X99 and Y00-Y09). This coding system is the most widely used framework for categorizing the circumstances of an injury and includes the intent and mechanism of injury.^18^

### Risk Factors

The main risk factor was immigrant status (nonimmigrant vs immigrant) determined based on the presence or absence of an Immigration, Refugees and Citizenship Canada Permanent Resident Database record. Within-immigrant risk factors included visa class (refugee vs nonrefugee), time in Canada since immigration (recent: 0-5 years; intermediate: 6-10 years;and long-term: >10 years), and region and country of origin (based on the country of birth). We included age, sex, neighborhood income quintile, and rurality as covariates.

### Statistical Analysis

Data were analyzed from April 13, 2017, to January 6, 2020. We described baseline characteristics of the cohort using frequencies and percentages. We calculated the total number of assaults overall and by mechanism during the 9-year study period and annual injury rates per 100 000 person-years directly standardized by age and sex using 2006 census estimates.

Multivariable modified Poisson regression models with generalized estimating equations to account for multiple events within the same individual were built to estimate the relative risk (RR) with 95% CI of assault-related injury by immigrant status. We produced an overall model including nonimmigrants and immigrants and stratified models separately with nonimmigrants and immigrants, adjusting for age, sex, neighborhood income, and rurality. We then conducted analyses to estimate the risk of experiencing assault for various immigration characteristics, with separate models run for visa class, time since immigration, and region of origin, adjusting for age, sex, neighborhood income, and rurality. Country-specific risk-adjusted rates were achieved by calculating the overall crude rate in nonimmigrants, country-specific crude rates, and estimated rates based on the modified Poisson model on the risk of assault in nonimmigrants; the 95% CIs of the risk-adjusted rates were then calculated using the bootstrapping method^19^ with unrestricted random sampling and replacement for 250 occurrences. Finally, we used the funnel plot method^20^ to compare country-level, risk-adjusted rates of assault with the observed nonimmigrant rate to identify outliers; the funnel plot control limits were calculated using a Poisson distribution. All analyses were conducted using SAS Enterprise Guide, version 6.1 (SAS Institute Inc).

## Results

We included 22 969 443 person-years (20 012 091 nonimmigrant and 2 957 352 immigrant; 51.3% male and 48.7% female) in our analyses (eFigure in the Supplement and Table 1). Immigrants had the largest proportion (1 288 711 [43.6%] vs 6 794 537 [34.0%]) in the oldest group (20-24 years), and there were similar proportions of male and female individuals in the immigrant (51.4% male and 48.6% female) and nonimmigrant (51.2% male and 48.8% female) groups. Immigrants had the largest proportion living in the lowest income quintile (30.5% among immigrants vs 18.2% among nonimmigrants). Almost all immigrants (98.9%) lived in urban settings in contrast to 87.7% of nonimmigrants. Among immigrants, 17.9% were refugees, 29.0% had been in Canada for less than 6 years, and 37.1% had been in Canada for more than 10 years. The largest source regions included South Asia (25.4%), East Asia and the Pacific (22.6%), the Middle East (12.9%), and the United States, United Kingdom, and Western Europe (10.6%).

**Table 1.** Cohort of Immigrant and Nonimmigrant Youth in Ontario, Canada, 2008-2016

Characteristic	Person-Years, No. (%) (N = 22 969 443)^a^	
	Nonimmigrants	Immigrants
Overall	20 012 091 (87.1)	2 957 352 (12.9)
Age, y		
10-14	6 423 847 (32.1)	677 833 (22.9)
15-19	6 793 707 (33.9)	990 808 (33.5)
20-24	6 794 537 (34.0)	1 288 711 (43.6)
Sex		
Female	9 757 813 (48.8)	1 437 599 (48.6)
Male	10 254 278 (51.2)	1 519 753 (51.4)
Neighborhood income quintile		
1 (low)	3 645 493 (18.2)	900 854 (30.5)
2	3 673 058 (18.4)	627 988 (21.2)
3	3 964 977 (19.8)	572 181 (19.3)
4	4 226 654 (21.1)	508 996 (17.2)
5 (high)	4 501 909 (22.5)	347 333 (11.7)
Rurality		
Urban	17 546 874 (87.7)	2 924 256 (98.9)
Rural	2 465 217 (12.3)	33 096 (1.1)
Immigrant class		
Nonrefugee	NA	2 428 834 (82.1)
Refugee	NA	528 518 (17.9)
Duration of residence		
Recent	NA	858 375 (29.0)
Intermediate	NA	1 001 948 (33.9)
Long term	NA	1 097 029 (37.1)
Region of origin		
United States, United Kingdom, or Western Europe	NA	313 654 (10.6)
East Asia and Pacific	NA	668 974 (22.6)
South Asia	NA	751 511 (25.4)
Eastern Europe or Central Asia	NA	268 252 (9.1)
Africa	NA	245 965 (8.3)
Middle East	NA	381 737 (12.9)
South America	NA	134 068 (4.5)
Central America	NA	192 357 (6.5)
Missing	NA	834 (0.03)

Abbreviation: NA, not applicable.

^a^ Percentages have been rounded and may not total 100.

During the 9-year study period, nonimmigrants experienced 110 936 assaults (549.0 [95% CI, 545.7-552.2] per 100 000 person-years), and immigrants experienced 9654 assaults (280.2 [95% CI, 305.4-321.0] per 100 000 person-years), including 225.0 (95% CI, 219.4-230.7) per 100 000 person-years in nonrefugee immigrant youth, and 525.4 (95% CI, 507.2-544.1) per 100 000 person-years in refugee immigrant youth (Table 2). In adjusted models, immigrants had less than half the risk of experiencing assault compared with nonimmigrants (adjusted RR [aRR], 0.49 [95% CI, 0.47-0.51]) (Table 3). Male nonimmigrants had higher rates of experiencing assault than female nonimmigrants (male: 779.1 [95% CI, 773.7-784.5] per 100 000 person-years; female: 309.1 [95% CI, 305.7-312.6] per 100 000 person-years; aRR, 2.54 [95% CI, 2.50-2.57]). Among immigrants, male youths had a higher rate of assault (436.9 [95% CI, 427.0-446.9] per 100 000 person-years) than female youths (117.1 [95% CI, 111.9-122.5] per 100 000 person-years) with risk of experiencing assault in male immigrants more than 4 times that of female immigrants (aRR, 3.78 [95% CI, 3.58-4.00]). Most assaults were experienced by the oldest youths, with rates as high as 863.9 (95% CI, 852.0-871.0) per 100 000 person-years in nonimmigrants aged 20 to 24 years and 488.2 (95% CI, 476.2-500.4) per 100 000 person-years in immigrants from that same age group. Risk of experiencing assault was higher in nonimmigrants from rural areas (aRR, 1.30 [95% CI, 1.28-1.32]) and not different between urban and rural immigrants.

**Table 2.** Frequency and Age-Sex Standardized Assault Rates by Immigration Status and Sociodemographic Characteristics in Ontario, Canada, 2008-2016

Characteristic	Nonimmigrants		Immigrants	
	No. of Assaults	Rate per 100 000 Person-Years (95% CI)	No. of Assaults	Rate per 100 000 Person-Years (95% CI)
Overall	110 936	549.0 (545.7-552.2)	9654	280.2 (305.4-321.0)
Age (sex standardized)				
10-14	7782	121.1 (118.3-123.7)	367	53.8 (48.5-59.6)
15-19	44 419	652.9 (646.8-659.0)	3010	301.4 (390.7-312.3)
20-24	58 735	863.9 (852.0-871.0)	6277	488.2 (476.2-500.4)
Sex (age standardized)				
Female	30 404	309.1 (305.7-312.6)	1950	117.1 (111.9-122.5)
Male	80 532	779.1 (773.7-784.5)	7704	436.9 (427.0-446.9)
Neighborhood income quintile				
1 (low)	33 250	886.2 (876.7-895.8)	3662	356.4 (344.7-368.3)
2	22 929	611.3 (603.4-619.3)	2077	277.7 (265.7-290.2)
3	19 910	499.7 (492.8-506.7)	1663	246.3 (234.4-258.6)
4	18 210	433.6 (427.3-440.0)	1370	230.9 (218.7-243.6)
5 (high)	16 637	367.2 (361.7-372.9)	882	218.1 (203.8-233.1)
Rurality				
Urban	93 110	526.6 (523.2-530.0)	9541	279.9 (274.2-285.6)
Rural	17 826	706.7 (696.4-717.2)	113	308.8 (253.2-373.1)
Immigrant class				
Nonrefugee	NA	NA	6320	225.0 (219.4-230.7)
Refugee	NA	NA	3334	525.4 (507.2-544.1)
Duration of residence				
Recent	NA	NA	2323	275.2 (264.1-286.6)
Intermediate	NA	NA	2852	277.4 (267.4-287.8)
Long term	NA	NA	4479	273.7 (264.9-282.6)
Region of origin				
United States/United Kingdom/Western Europe	NA	NA	1008	296.4 (278.3-315.4)
East Asia and Pacific	NA	NA	905	113.5 (106.0-121.3)
South Asia	NA	NA	1769	207.8 (198.2-217.7)
Eastern Europe/Central Asia	NA	NA	1228	378.2 (356.3-401.0)
Africa	NA	NA	1291	455.3 (430.4-481.1)
Middle East	NA	NA	1474	344.4 (326.9-362.5)
South America	NA	NA	656	401.5 (370.6-434.3)
Central America	NA	NA	1317	552.2 (521.0-584.8)
Missing	NA	NA	6	719.4 (168.7-1008.4)

Abbreviation: NA, not applicable.

**Table 3.** Adjusted RRs of Assault-Related Injuries by Immigrant Status and Sociodemographic Characteristics, 2008-2016

Characteristic	aRR (95% CI)		
	Overall Model	Stratified Models	
		Nonimmigrants Only	Immigrants Only
Immigrant status			
Nonimmigrant	1 [Reference]	NA	NA
Immigrant	0.49 (0.47-0.51)	NA	NA
Age, y			
10-14	1 [Reference]	1 [Reference]	1 [Reference]
15-19	5.26 (4.97-5.55)	5.38 (5.25-5.52)	5.37 (4.79-6.02)
20-24	6.90 (6.53-7.29)	6.96 (6.79-7.14)	8.49 (7.59-9.50)
Sex			
Female	1 [Reference]	1 [Reference]	1 [Reference]
Male	2.60 (2.52-2.68)	2.54 (2.50-2.57)	3.78 (3.58-4.00)
Neighborhood income quintile			
1 (low)	2.42 (2.32-2.53)	2.38 (2.33-2.43)	1.58 (1.46-1.72)
2	1.69 (1.61-1.77)	1.65 (1.62-1.69)	1.26 (1.16-1.37)
3	1.34 (1.27-1.40)	1.35 (1.32-1.38)	1.13 (1.03-1.23)
4	1.25 (1.19-1.31)	1.18 (1.15-1.20)	1.06 (0.97-1.16)
5 (high)	1 [Reference]	1 [Reference]	1 [Reference]
Rurality			
Urban	1 [Reference]	1 [Reference]	1 [Reference]
Rural	1.28 (1.23-1.33)	1.30 (1.28-1.32)	1.07 (0.87-1.31)

Abbreviation: aRR, adjusted rate ratio; NA, not applicable.

Table 4 shows the aRRs for assault by immigration characteristics. Compared with nonimmigrants, refugees (aRR, 0.82 [95% CI, 0.76-0.89]) and nonrefugees (aRR, 0.41 [95% CI, 0.38-0.43]) had a lower risk of experiencing assault. Risk of experiencing assault was not different with duration of time in Canada and was lower in immigrants across all regions of origin except for those from Central America, with lowest rates experienced by immigrants from East Asia (aRR, 0.23 [95% CI, 0.19-0.26]) and South Asia (aRR, 0.33 [95% CI, 0.30-0.37]) (Table 4). Of all the regions of origin, immigrants from Central America had the highest rates of experiencing assault (552.2 [95% CI, 521.0-584.8] per 100 000 person-years; aRR, 0.91 [95% CI, 0.80-1.03]) compared with nonimmigrants. Older age (oldest vs youngest aRR, 6.90 [95% CI, 6.53-7.29]), male sex (aRR, 2.60 [95% CI, 2.52-2.68]), and low income (aRR, 2.42 [95% CI, 2.32-2.53]) were associated with violent injury risk.

**Table 4.** Adjusted RRs of Assault-Related Injuries by Immigrant Characteristics, 2008-2016^a^

Characteristic	aRR (95% CI)
Model 1: visa class	
Nonimmigrant	1 [Reference]
Nonrefugee immigrants	0.41 (0.38-0.43)
Refugee immigrants	0.82 (0.76-0.89)
Model 2: duration of residence	
Nonimmigrant	1 [Reference]
Recent	0.49 (0.44-0.53)
Intermediate	0.46 (0.42-0.50)
Longer term	0.52 (0.48-0.55)
Model 3: region of origin	
Nonimmigrant	1 [Reference]
United States/United Kingdom/Western Europe	0.59 (0.51-0.68)
East Asia	0.23 (0.19-0.26)
South Asia	0.33 (0.30-0.37)
Eastern Europe/Central Asia	0.71 (0.62-0.80)
Africa	0.76 (0.67-0.87)
Middle East	0.57 (0.50-0.65)
South America	0.67 (0.56-0.80)
Central America	0.91 (0.80-1.03)

Abbreviation: aRR, adjusted rate ratio.

^a^ Includes 22 969 443 person-years.

The Figure shows the funnel plot of country-specific, risk-adjusted rates of experiencing assault. Only those countries of origin with at least 20 individuals assaulted are shown, and specific rates are in eTable 1 in the Supplement. Immigrants from every country had rates no different than or lower than the nonimmigrant rate except for immigrants from Somalia, where rates were 712.0 (95% CI, 639.3-805.3) per 100 000 person-years. Immigrants from all large-volume countries (>75 000 person-years) had rates lower than nonimmigrants, including immigrants from India, China, Pakistan, Philippines, Sri Lanka, and the United States, except for Jamaica and Iran, whose rates were not different than those of Canadian-born individuals.

**Figure.**
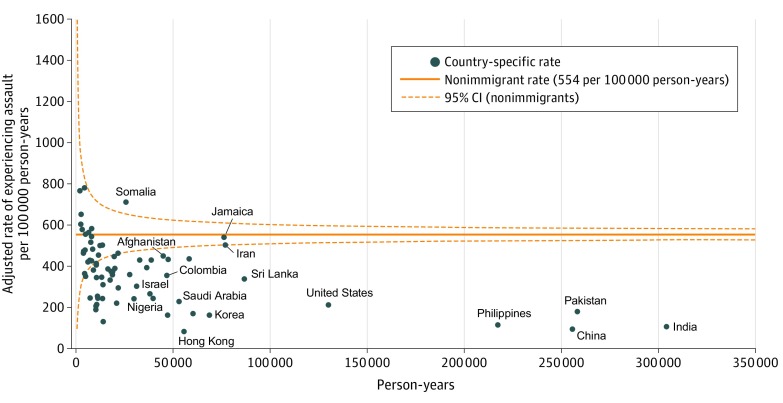
Rates of Assault by Country of Immigration Rates were adjusted for age, sex, neighborhood income, and rurality.

The mechanism of assault by immigrant status is shown in eTable 2 in the Supplement. Most assaults were from being struck (79.9%) followed by being cut (5.9%). Immigrants had lower or similar rates of assaults from all causes except for firearm assaults (5.2 [95% CI, 4.4-6.1] per 100 000 person-years vs 3.3 [95% CI, 3.1-3.6] per 100 000 person-years) and those caused by cutting or piercing (31.3 [95% CI, 29.4-33.4] per 100 000 person-years vs 30.6 [95% CI, 29.9-31.4] per 100 000 person-years), for which rates were higher among immigrants.

## Discussion

In this population-based study, we demonstrated that the risk of experiencing assault among immigrant youth was 51% lower than in Canadian-born youth. Rates of assault were particularly low among youth from Canada’s largest intake countries, including Pakistan, China, India, Philippines, the United States, and Sri Lanka. We found an increased risk of experiencing assault in refugee compared with nonrefugee immigrants but no change in this risk with increasing time since migration to Canada. There was substantial variability in the risk of experiencing assault by region and country of origin, with the lowest rates among those from South and East Asia and the highest among those from Central America and Africa. Importantly, we demonstrated high rates of experiencing assault among Somali immigrant youth.

Comparing rates of violence experienced by immigrants in the present study, which includes violence experienced from peers, friends, relations, and strangers, with rates in other jurisdictions is difficult given the paucity of published studies on those who experience and survive violence that do not rely on self-report or only focus on intimate partner violence or homicides. One Swiss study in adolescents who experienced child maltreatment, including physical assault,^21^ found that the prevalence of assault was lowest in native Swiss, higher in Western immigrants, and highest in non-Western immigrants. The investigators found that, after adjusting for other risk factors, a family background of migration was the strongest risk factor for physical abuse.^21^ In Italy, foreign-born youth were more likely to perpetrate and experience homicide compared with Italian-born youth^22^; in Spain, foreign-born women were more likely to report experiencing violence than nonimmigrants.^23^ In the Netherlands, immigrant children were overrepresented in families who had witnessed prior violence (child maltreatment, neglect, or domestic violence) or homicide in their home.^24^ In contrast to studies of immigrants in Europe, most reports on individuals who experience violence in North America suggest immigrants are at lower risk than nonimmigrants. In Canada, adult immigrants reported experience of violent assault at almost half the rate of nonimmigrants (68 vs 116 per 1000 population).^25^ Wheeler et al^26^ reported that foreign-born adults in the United States have self-reported rates of experiencing violence not different from those of US-born adults. However, they also reported that, for immigrants entering the United States as youth, the prevalence of experiencing violence declined with longer residency in the United States.^26^

Rates of assault may be lower in immigrant youth to Canada for several reasons. Canada accepts relatively high proportions of immigrants in economic and family classes in which socioeconomic disadvantage, language proficiency, exposure to trauma, and forced migration may be lower than that among immigrants in other jurisdictions.^4^ However, refugees to Canada experienced relatively low rates of assault. This finding suggests that, although prior experiences, including violence, conflict, and forced migration, may contribute to some extent, other factors, such as family cohesion, hope, opportunity, settlement supports, and living in urban centers with high-density immigrant communities (associated with reduced crime and better health outcomes), may contribute to our findings.^8,27,28,29^

We found that with increasing time since migration, there was no change in risk of experiencing violence. Other studies have found that time since migration is associated with increasing perpetration of violence, increasing substance use, and increasing intimate partner violence.^10,30^ Instead, our findings demonstrate that immigrants maintain their health advantage with time in Canada. Refugees also had higher rates of experiencing assault compared with nonrefugees. Alink et al^31^ have shown higher rates of child maltreatment in refugee compared with nonrefugee families. Recent immigrants and refugees may be vulnerable to relative poverty and intimate partner violence, may have limited support systems with changing family dynamics, and may have new gender roles.^6,32^ However, their rates of experiencing violence are lower than those of native-born youths.

Country-specific rates of assault in immigrants to Canada show low rates from each of Canada’s major source countries. However, violence experienced by Somali youth, most of whom come as refugees (76%), was disproportionately high compared with violence experienced by other immigrant groups. This violence may undermine their capacity to rebuild their lives in Canada and has been shown to perpetuate through generations, in part due to barriers to equitable opportunities in education and participation in the labor market.^33,34^ Our findings highlight that policy makers, clinicians, and settlement support workers need to address the unique sources of risk specific to the Somali immigrant population, including but not limited to the compounding effects of racism and Islamophobia, and work to identify institutional barriers at the root of violence.^33,35^

Our study included a large sample size with almost complete coverage of the population of youth. Our findings include only substantiated assaults that are complimentary to studies on self-report and may have reporting bias, especially among immigrants hesitant to report experiencing assault. We included a large number of immigrants from all regions of the globe with detailed immigration data that allowed better understanding of immigration-specific risk factors for violent injury.

### Limitations

A limitation of this study is that some important factors are unknown. These factors include the circumstances contributing to injury events (eg, perpetrator, gang involvement vs family violence) and some demographic information (eg, family educational level and language proficiency), both of which may have helped to further understand injury risk. Care seeking between immigrants and nonimmigrants may differ; thus, injuries may be underestimated among immigrants, particularly for more minor physical injuries. Immigration data was limited to permanent residents and did not include temporary (eg, foreign students or workers) or undocumented immigrants.

## Conclusions

The low relative rates of assault among immigrants, in particular the almost universally equal or lower rates by country of origin, suggest that the Canadian immigrant settlement support system and cultural factors may be protective against the risk of experiencing assault. Results highlight a need for increased attention to immigrants from Somalia for whom vulnerabilities may be inadequately addressed and for whom settlement supports and opportunities for success may be insufficient.

## Notes

Supplement. **eFigure.** Flowsheet of Inclusion and Exclusion Criteria **eTable 1.** Country-Specific Numbers and Rates of Experiencing Assault **eTable 2.** Mechanism of Assault-Related Injury by Immigrant Status, 2008-2016

## References

[1] Kyu HH, Pinho C, Wagner JA, ; Global Burden of Disease Pediatrics Collaboration Global and national burden of diseases and injuries among children and adolescents between 1990 and 2013: findings from the Global Burden of Disease 2013 Study. JAMA Pediatr. 2016;170(3):-. doi:10.1001/jamapediatrics.2015.4276 26810619 PMC5076765

[2] Sminkey L World report on child injury prevention. Inj Prev. 2008;14(1):69. doi:10.1136/ip.2007.018143 18245322

[3] Perreault S, Brennan S Criminal Victimization in Canada: 2009. Ottawa, ON: Statistics Canada; 2010.

[4] Facts & figures 2015: immigration overview—permanent residents—annual IRCC updates. https://open.canada.ca/data/en/dataset/2fbb56bd-eae7-4582-af7d-a197d185fc93?_ga=2.177922405.1187136379.1499268237-455880055.1499268237. Government of Canada website. Published 2015. Accessed July 6, 2017.

[5] United Nations Department of Economic and Social Affairs, Population Division. International migration report 2017: highlights. https://www.un.org/en/development/desa/population/migration/publications/migrationreport/docs/MigrationReport2017_Highlights.pdf. Published 2017. Accessed July 8, 2019.

[6] Okeke-Ihejirika P, Yohani S, Muster J, Ndem A, Chambers T, Pow V A scoping review on intimate partner violence in Canada’s immigrant communities [published online September 3, 2018]. Trauma Violence Abuse. doi:10.1177/1524838018789156 30176768

[7] Grace BL, Bais R, Roth BJ The violence of uncertainty: undermining immigrant and refugee health. N Engl J Med. 2018;379(10):904-905. doi:10.1056/NEJMp1807424 30184446

[8] Antunes MJL, Ahlin EM Minority and immigrant youth exposure to community violence: the differential effects of family management and peers [published online February 1, 2018]. J Interpers Violence. doi:10.1177/0886260518755491 29400148

[9] Chavez JM, Griffiths E Neighborhood dynamics of urban violence: understanding the immigration connection. Homicide Stud. 2009;13(3). doi:10.1177/1088767909337701 24222722 PMC3821546

[10] Almeida J, Johnson RM, McNamara M, Gupta J Peer violence perpetration among urban adolescents: dispelling the myth of the violent immigrant. J Interpers Violence. 2011;26(13):2658-2680. doi:10.1177/0886260510388288 21156691 PMC3123437

[11] Ewing W, Martinez D, Rumbaut R *The Criminalization of Immigration in the United States.* Washington, DC: American Immigration Council Special Report; 2015.

[12] Rumbaut R, Ewing W The Myth of Immigrant Criminality and the Paradox of Assimilation: Incarceration Rates Among Native and Foreign-Born Men. Washington, DC: Immigration Policy Center; 2007.

[13] Walsh SD, De Clercq B, Molcho M, The relationship between immigrant school composition, classmate support and involvement in physical fighting and bullying among adolescent immigrants and non-immigrants in 11 countries. J Youth Adolesc. 2016;45(1):1-16. doi:10.1007/s10964-015-0367-0 26502194

[14] Pottie K, Dahal G, Georgiades K, Premji K, Hassan G Do first generation immigrant adolescents face higher rates of bullying, violence and suicidal behaviours than do third generation and native born? J Immigr Minor Health. 2015;17(5):1557-1566. doi:10.1007/s10903-014-0108-6 25248622 PMC4562994

[15] Foster H, Brooks-Gunn J Neighborhood, family and individual influences on school physical victimization. J Youth Adolesc. 2013;42(10):1596-1610. doi:10.1007/s10964-012-9890-4 23263822 PMC3732577

[16] Chiu M, Lebenbaum M, Lam K, Describing the linkages of the immigration, refugees and citizenship Canada Permanent Resident Data and Vital Statistics Death Registry to Ontario’s administrative health database. BMC Med Inform Decis Mak. 2016;16(1):135. doi:10.1186/s12911-016-0375-3 27769227 PMC5073414

[17] Kralj B Measuring Rurality—RIO2008 BASIC: Methodology and Results. Toronto, ON: Ontario Medical Association Economics Department; 2009.

[18] Holder Y, Peden M, Krug E Injury Surveillance Guidelines. Geneva, Switzerland: World Health Organization; 2001.

[19] Iezzoni LI Risk Adjustment for Measuring Healthcare Outcomes. 4th ed. Chicago, IL: Health Administration Press; 2012.

[20] Manktelow BN, Seaton SE, Evans TA Funnel plot control limits to identify poorly performing healthcare providers when there is uncertainty in the value of the benchmark. Stat Methods Med Res. 2016;25(6):2670-2684. doi:10.1177/0962280214530281 24742429

[21] Schick M, Schönbucher V, Landolt MA, Child maltreatment and migration: a population-based study among immigrant and native adolescents in Switzerland. Child Maltreat. 2016;21(1):3-15. doi:10.1177/1077559515617019 26590238

[22] Terranova C, Zen M, Maguolo N, Cirillo T, Montisci M Underage victims and perpetrators of murder in Italy: 2007-2015. J Forensic Leg Med. 2018;59:39-44. doi:10.1016/j.jflm.2018.08.002 30130701

[23] Colorado-Yohar S, Tormo MJ, Salmerón D, Dios S, Ballesta M, Navarro C Violence reported by the immigrant population is high as compared with the native population in southeast Spain. J Interpers Violence. 2012;27(16):3322-3340. doi:10.1177/0886260512441260 22809817

[24] Alisic E, Groot A, Snetselaar H, Stroeken T, van de Putte E Children bereaved by fatal intimate partner violence: a population-based study into demographics, family characteristics and homicide exposure. PLoS One. 2017;12(10):e0183466. doi:10.1371/journal.pone.0183466 28976977 PMC5627890

[25] Gannon M, Mihorean K Criminal Victimization in Canada, 2004. Ottawa, Canada: Statistics Canada; 2005.

[26] Wheeler K, Zhao W, Kelleher K, Stallones L, Xiang H Immigrants as crime victims: experiences of personal nonfatal victimization. Am J Ind Med. 2010;53(4):435-442. doi:10.1002/ajim.20820 20196094

[27] Eschbach K, Ostir GV, Patel KV, Markides KS, Goodwin JS Neighborhood context and mortality among older Mexican Americans: is there a barrio advantage? Am J Public Health. 2004;94(10):1807-1812. doi:10.2105/AJPH.94.10.1807 15451754 PMC1448538

[28] Rossiter M, Rossiter K Immigrant Youth and Crime: Stakeholder Perspectives on Risk and Protective Factors. Edmonton, AB: Prairie Metropolis Centre; 2009.

[29] Sabina C, Cuevas CA, Schally JL The effect of immigration and acculturation on victimization among a national sample of Latino women. Cultur Divers Ethnic Minor Psychol. 2013;19(1):13-26. doi:10.1037/a0030500 23148902

[30] Gupta J, Acevedo-Garcia D, Hemenway D, Decker MR, Raj A, Silverman JG Intimate partner violence perpetration, immigration status, and disparities in a community health center-based sample of men. Public Health Rep. 2010;125(1):79-87. doi:10.1177/003335491012500111 20402199 PMC2789819

[31] Alink LR, Euser S, van Ijzendoorn MH, Bakermans-Kranenburg MJ Is elevated risk of child maltreatment in immigrant families associated with socioeconomic status? evidence from three sources. Int J Psychol. 2013;48(2):117-127. doi:10.1080/00207594.2012.734622 23597011

[32] Stewart M, Dennis CL, Kariwo M, Challenges faced by refugee new parents from Africa in Canada. J Immigr Minor Health. 2015;17(4):1146-1156. doi:10.1007/s10903-014-0062-3 24989494

[33] Aden MIA, Rayale S, Abokor L Another Day, Another Janazah: An Investigation Into Violence, Homicide and Somali-Canadian Youth in Ontario. Toronto, ON: Youthleaps; 2018.

[34] Gele AA, Pettersen KS, Torheim LE, Kumar B Health literacy: the missing link in improving the health of Somali immigrant women in Oslo. BMC Public Health. 2016;16(1):1134. doi:10.1186/s12889-016-3790-6 27809815 PMC5093985

[35] Betancourt TS, Abdi S, Ito BS, Lilienthal GM, Agalab N, Ellis H We left one war and came to another: resource loss, acculturative stress, and caregiver-child relationships in Somali refugee families. Cultur Divers Ethnic Minor Psychol. 2015;21(1):114-125. doi:10.1037/a0037538 25090142 PMC4315611

