# Supplementary material for: Association of Immigrant and Refugee Status With Risk Factors for Exposure to Violent Assault Among Youths and Young Adults in Canada
Source: JAMA Netw Open. 2020 Mar 4;3(3):e200375. doi: 10.1001/jamanetworkopen.2020.0375 (PMC7057130; doi:10.1001/jamanetworkopen.2020.0375)
Supplement: Supplement. — eFigure. Flowsheet of Inclusion and Exclusion Criteria eTable 1. Country-Specific Numbers and Rates of Experiencing Assault eTable 2. Mechanism of Assault-Related Injury by Immigrant Status, 2008-2016 [file jamanetwopen-3-e200375-s001.pdf]

Supplementary Online Content

Saunders NR, Guan J, Macpherson A, Lu H, Guttmann A. Association of immigrant and refugee status with risk factors for exposure to violent assault among youths and young adults in Canada. *JAMA Netw Open*. 2020;3(3):e200375. doi:10.1001/jamanetworkopen.2020.0375

eFigure. Flowsheet of Inclusion and Exclusion Criteria

eTable 1. Country-Specific Numbers and Rates of Experiencing Assault

eTable 2. Mechanism of Assault-Related Injury by Immigrant Status, 2008-2016

This supplementary material has been provided by the authors to give readers additional information about their work.

©2020 Saunders NR et al. *JAMA Network Open*.

eFigure. Flowsheet of Inclusion and Exclusion Criteria

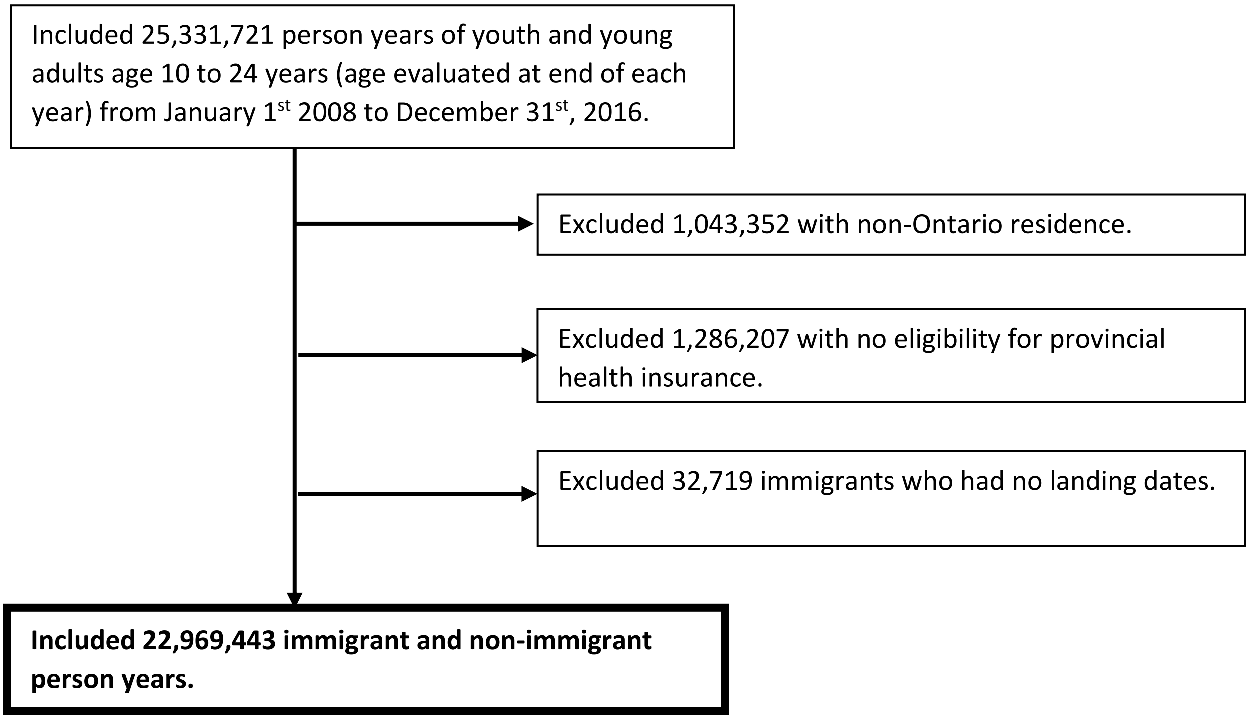

©2020 Saunders NR et al. *JAMA Network Open*.

eTable 1. Country-Specific Numbers and Rates of Experiencing Assault<br>Only top 30 source countries or those with greater than 100 events listed.			
**Country**	**Number of assaults**	**Population**	**Adjusted rate (per 100,00 population) (95% CI)**
India	379	304135	106.8 (94.7, 117.8)
Pakistan	541	258163	180.6 (163.2, 196.3)
China	298	255619	95.6 (85.9, 106.4)
Philippines	313	216973	115 (101.4, 130.7)
United States of America	243	130060	212.9 (188.2, 241.3)
Sri Lanka	413	86460	338.8 (304.9, 380.5)
Iran	448	77014	503.4 (455.1, 552.9)
Jamaica	586	76226	541.1 (490.2, 596.6)
Korea	129	68686	162.5 (132.9, 192.1)
United Kingdom and Colonies	236	64854	363.7 (317.0, 416.4)
United Arab Emirates	105	60129	169.4 (138.9, 205.9)
Iraq	334	58367	436.6 (389.5, 479.4)
Hong Kong	60	55619	83.3 (62.4, 103.2)
Saudi Arabia	143	53100	228.9 (192.4, 269.0)
Russia	234	47529	432.9 (377.3, 492.9)
Bangladesh	99	47120	163.3 (130.3, 202.7)
Colombia	206	46662	354.9 (312.5, 405.2)
Afghanistan	308	44701	449.1 (390.2, 500.8)
Egypt	96	39499	243.4 (193.6, 300.1)
Ukraine	204	38767	430.7 (377.8, 504.7)
Romania	112	37962	266.4 (213.7, 325.3)
Guyana	205	36319	393.1 (331.1, 462.2)
Yugoslavia	184	32712	429.7 (349.2, 498.9)
Israel	79	31124	302.6 (221.8, 376.9)
Nigeria	80	29755	243.3 (176.7, 299.4)
Poland	137	27493	359.1 (298.2, 421.0)
Somalia	329	25583	712.0 (639.3, 805.3)
Kuwait	78	21712	295.6 (240.7, 367.4)
Trinidad and Tobago	136	21317	463.0 (374.4, 538.9)
Mexico	104	19676	447.5 (351.8, 541.5)
Sudan	100	13557	503.0 (407.0, 597.3)

©2020 Saunders NR et al. *JAMA Network Open*.

eTable 2. Mechanism of Assault-Related Injury by Immigrant Status, 2008-2016

	Non-immigrants		Immigrants	
	**Number of injuries**	**Crude rate per 100 000 person years (95% CI)**	**Number of injuries**	**Crude rate per 100 000 person years (95% CI)**
Overall	110936	554.3 (551.1, 557.6)	9654	326.4 (320.0, 333.0)
Cut/pierce	6133	30.6 (29.9, 31.4)	926	31.3 (29.4, 33.4)
Fall	90	0.5 (0.4, 0.6)	6	0.2 (0.1, 0.5)
Firearm	670	3.3 (3.1, 3.6)	154	5.2 (4.4, 6.1)
Poisoning	477	2.4 (2.2, 2.6)	38	1.3 (0.9, 1.8)
Struck by/against	89117	445.3 (442.4, 448.3)	7220	244.1 (238.6, 249.8)
Other	14449	72.2 (71.0, 73.4)	1310	44.3 (42.0, 46.8)

©2020 Saunders NR et al. *JAMA Network Open*.
